# Dual Role of Mo_6_S_8_ in Polysulfide Conversion and Shuttle for Mg–S Batteries

**DOI:** 10.1002/advs.202104605

**Published:** 2022-01-09

**Authors:** Liping Wang, Piotr Jankowski, Christian Njel, Werner Bauer, Zhenyou Li, Zhen Meng, Bosubabu Dasari, Tejs Vegge, Juan Maria García Lastra, Zhirong Zhao‐Karger, Maximilian Fichtner

**Affiliations:** ^1^ Helmholtz Institute Ulm (HIU) Electrochemical Energy Storage Helmholtzstrasse 11 Ulm D‐89081 Germany; ^2^ Department of Energy Conversion and Storage Technical University of Denmark Kongens Lyngby 2800 Denmark; ^3^ Faculty of Chemistry Warsaw University of Technology Warsaw 00664 Poland; ^4^ Institute for Applied Materials‐Energy Storage Systems (IAM‐ESS) and Karlsruhe Nano Micro Facility (KNMF) Karlsruhe Institute of Technology (KIT) Hermann‐von‐Helmholtz‐Platz 1 Eggenstein‐Leopoldshafen D‐76344 Germany; ^5^ Institute of Nanotechnology (INT) Karlsruhe Institute of Technology (KIT) Hermann‐von‐Helmholtz Platz 1 Eggenstein‐Leopoldshafen D‐76344 Germany

**Keywords:** catalytic effect, Chevrel phase Mo_6_S_8_, density functional theory calculations, functional separator, magnesium–sulfur batteries, polysulfide shuttle

## Abstract

Magnesium–Sulfur batteries are one of most appealing options among the post‐lithium battery systems due to its potentially high energy density, safe and sustainable electrode materials. The major practical challenges are originated from the soluble magnesium polysulfide intermediates and their shuttling between the electrodes, which cause high overpotentials, low sulfur utilization, and poor Coulombic efficiency. Herein, a functional Mo_6_S_8_ modified separator is designed to effectively address these issues. Both the experimental results and density functional theory calculations show that the electrochemically active Mo_6_S_8_ layer has a superior adsorption capability of polysulfides and simultaneously acts as a mediator to accelerate the polysulfide conversion kinetics. Remarkably, the magnesium–sulfur cell assembled with the functional separator delivers a high specific energy density (942.9 mA h g^−1^ in the 1st cycle) and can be cycled at 0.2 C for 200 cycles with a Coulombic efficiency of 96%. This work demonstrates a new design concept toward high‐performance metal–sulfur batteries.

## Graphical abstract

A Mo6S8‐modified separator is utilized to suppress the polysulfide shuttle in Mg–S batteries. Based on experimental investigations and density functional theory calculations, the separator shows a superior adsorption capability of polysulfide and a catalytic effect on the polysulfide conversion reactions. Mg–S cells in both coin and pouch cell configurations can achieve high reversible capacities with extended cycle life.

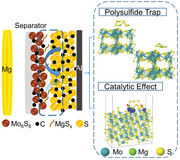

## 1 Introduction

The rapidly growing global market for electric vehicles and grid‐scale electricity storage gives rise to concerns about the long‐term availability of certain raw materials such as cobalt, nickel, and graphite,^[^ ^1^ ^]^ which are essential components in current commercial lithium ions batteries (LIBs).^[^ ^2^ ^]^ In this regard, alternative high‐energy systems based on sustainable materials have gained increasing attention. Among various candidates, rechargeable magnesium batteries have emerged as attractive candidates because of the ideal features of metallic magnesium (Mg) as a metal anode. Mg metal has a low reduction potential (−2.356 V vs SHE) and a high theoretical volumetric capacity of 3832 mA h cm^−3^, which is considerably higher than that of Li in graphitic anodes (≈700 mA h cm^−3^), Li metal (2062 mA h cm^−3^) and sodium metal (1136 mA h cm^−3^).^[^ ^3^ ^]^ Furthermore, the cost of Mg metal is 30 times cheaper than Li metal.^[^ ^4^ ^]^ In 2000, Aurbach et al. have demonstrated a prototype system for rechargeable magnesium batteries with a Chevrel phased (CP) Mo_6_S_8_ cathode.^[^ ^5^ ^]^ With an open 3D framework, CP permits relatively fast reversible intercalation of Mg ions.^[^ ^6^ ^]^ Despite the good electrochemical performance, the limited specific energy (≈100 mA h g^−1^) of Mo_6_S_8_ cathode materials restricts their use for practical applications.^[^ ^7^ ^]^

Sulfur (S) cathodes have received increasing attention due to its high theoretical capacity (1675 mA h g^−1^), low toxicity, and abundance. Based on two‐electron redox reactions between sulfur cathode and Mg metal anode (Mg^2+^ + S + 2e^−^ ⇄ MgS, 1.77 V), magnesium–sulfur (Mg–S) battery possesses a higher theoretical volumetric capacity than lithium–sulfur (Li–S) batteries, making it a promising candidate for emerging energy storage markets.^[^ ^8^ ^]^ Nevertheless, similar to the Li–S battery chemistry, due to the known “shuttle” phenomena of polysulfide, Mg–S batteries suffer from continuous self‐discharge, rapid capacity decay and short cell life.^[^ ^3^, ^8^, ^9^ ^]^ Pioneer works have been devoted to tackling the problematics related to the dissolution and migration of polysulfide species in Mg–S batteries, including designing sulfur cathode architectures,^[^ ^10^ ^]^ electrolyte compositions,^[^ ^4^, ^11^ ^]^ and functional separators.^[^ ^12^ ^]^

Herein, we report a new approach to modify the separator by coating Chevrel phase Mo_6_S_8_ on a commonly used Celgard separator (CG), denoted as CG@CP, for Mg–S batteries. The Chevrel phase Mo_6_S_8_ has several unique properties. First, it is an electrochemically active material in the ether‐based electrolyte within the same voltage window as S_8_. Second, it has both high electronic and ionic conductivities. Third, from the density functional theory (DFT) calculations, it has a high affinity for polysulfides and may benefit for conversion reactions of the polysulfides. To clarify the function of the CG@CP separator, electrochemical investigations were carried out in Mg–S cells with a model sulfur cathode and magnesium borate‐based electrolyte. Comparison was evaluated between the cells with CG@CP, pristine CG, and carbon‐coated separator (CG@C), respectively. The combined experimental and theoretical studies revealed that the functional separator can suppress the polysulfide migration to the anode side and shows a catalytic effect on the transformation of polysulfides. In addition, Mg–S pouch cells with CG@CP were fabricated to verify its feasibility for practical implementations.

## 2 Results and Discussion

### 2.1 Structure and Morphology of CG@CP

**Figure 1:**
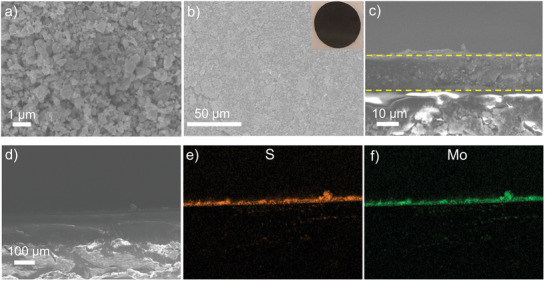
a) SEM image of Mo_6_S_8_ powder. b) SEM image of CG@CP (inset: the photograph of CG@CP). c–f) The cross‐sectional SEM images and EDS maps of CG@CP.

The diffractogram of Mo_6_S_8_ sample can be well indexed to standard Mo_3_S_4_ with rhombohedral structure as shown in Figure S1a of the Supporting Information. Besides, the weak reflection at ≈15° is corresponding to small amount of a MoS_2_ impurity. The scanning electron microscopy (SEM) and energy dispersive spectroscopy (EDS) maps in **Figure** **1**a and Figure S1b–d (Supporting Information) show that molybdenum and sulfur are uniformly distributed in the Mo_6_S_8_ particles. By casting Mo_6_S_8_/SuperP slurry onto the separator membrane, the CP‐modified separator was prepared. The corresponding SEM image and optical photo are shown in Figure 1b and the elemental distribution of the coated membrane was characterized with EDS (Figure S2, Supporting Information). The results clearly demonstrate that the granular Mo_6_S_8_ was coated on the membrane with a homogeneous distribution. Compared with the pristine CG as shown in Figure S3 of the Supporting Information, Mo_6_S_8_ particles fully covered the surface of CG and filled the pores of the separator. The corresponding cross‐sectional EDS maps of CG@CP are shown in Figure 1c–f, which manifest that the separator is uniformly coated by a CP film with an average thickness of ≈20 µm. Furthermore, the CG@CP separator possesses a good mechanical robustness and flexibility as there was no detectable delamination after repeated bending (Figure S4b, Supporting Information).

### 2.2 Polysulfides Anchoring Capability of Mo_6_S_8_

**Figure 2:**
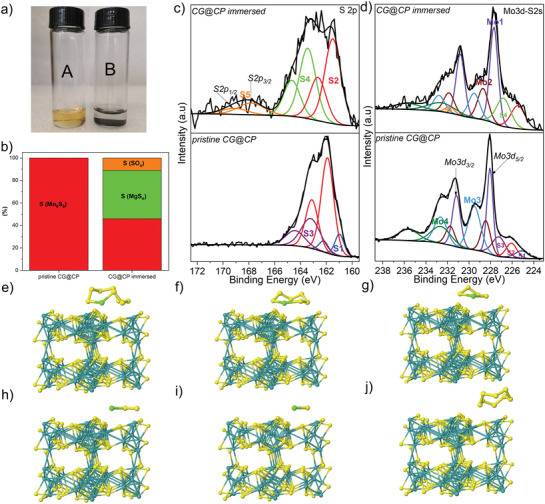
a) Digital photograph of the magnesium polysulfide solution before (A) and after the addition of Mo_6_S_8_ powder (B), respectively. b) Relative atomic percentages of the sulfur‐containing compounds on the surfaces of CG@CP before and after immersion, all the samples were measured after drying. c) S 2p spectra of pristine CG@CP and CG@CP immersed in MgS_n_ solution (upper). d) Mo 3d‐S 2s spectra of pristine CG@CP and CG@CP immersed in MgS_n_ solution (upper). The optimized geometries of e–i) MgS_n_ (n = 8, 6, 4, 2, 1) and j) S_8_ species adsorption at the Chevrel phase surface.

To evaluate the interaction between Mo_6_S_8_ and the dissolved magnesium polysulfide, an adsorption experiment was conducted using a magnesium polysulfide (MgS_n_) solution. The solution was prepared according to a reported procedure^[^ ^11c^ ^]^ and the corresponding photograph of the prepared MgS_n_ solution is presented in Figure S5b of the Supporting Information. A simple visual adsorption test was performed by dispersing the Mo_6_S_8_ powder in the MgS_n_ solution. As shown in **Figure** **2**a, it became colorless after the addition of Mo_6_S_8_ powder, which confirms an intrinsic trapping capability of Mo_6_S_8_ to the polysulfides. To further demonstrate the effect of modified separator on the suppression of polysulfide diffusion, two H‐type glass cells were assembled with a pristine CG separator and a CG@CP separator. The tetraglyme with and without magnesium polysulfide (MgS_n_) solutions were injected in the left and the right chamber, respectively. As shown in Figure S6 of the Supporting Information, in the H‐type cell with the pristine CG separator (right), the red‐brown polysulfides gradually migrated through the separator from left to right within 24 h. By contrast, there is no obvious polysulfide diffusion observed from the cell with CG@CP separator (left) even after 72 h, which confirms that the CG@CP can effectively block the polysulfide diffusion.

To gain deeper understanding of the polysulfide interaction with Mo_6_S_8_ coated separator, X‐ray photoelectron spectroscopy (XPS) was employed. S 2p and Mo 3d‐S 2s XPS spectra of the pristine CG@CP and CG@CP immersed in MgS_n_ solution (24 h) are presented in Figure 2c,d. The S 2p spectra have been fitted with 2p_3/2_–2p_1/2_ doublets separated by 1.2 eV with 2/1 intensity ratio due to spin–orbit coupling. The S5 (167.9–169.1 eV) and S4 (163.5–164.7 eV) doublets correspond to SO_x_ and MgS_n_ environments, respectively. After immersion, the signal of the polysulfide species (43% of sulfur signal) was detected on the separator, which hints at a relatively strong adsorption of the sulfur species onto Mo_6_S_8_ (Figure 2b). The slight shift (≈0.5 eV) toward the low binding energies of the characteristic peaks of Mo_6_S_8_ (S2, Mo1, Mo2, and Mo3) hints at the existence of a chemical interaction between the polysulfides and Mo_6_S_8_. Moreover, DFT calculations were used to determine the interaction between different magnesium polysulfides and the surface of the Chevrel phase material. Calculated interaction energies (Table S1, Supporting Information) indicate a strong adsorption of MgS_n_ at the surface of CP (Figure 2e–j), with the binding energies of more than 2 eV whereas the adsorption energies on carbon can hardly reach 1 eV (Figure S7, Supporting Information). Interestingly, upon magnesiation of CP, the intensity of the adsorption decreases slightly, but it is still very strong at all magnesiation levels and can be expected to prevent the migration of magnesium polysulfides to the anode.

### 2.3 Electrochemical Performance of Mg–S Batteries

To evaluate the influence of the coating layer on Mg‐ion transport properties, electrochemical impedance spectroscopy (EIS) was performed. The ionic conductivity was typically measured in a symmetrical stainless steel two‐electrode device (Figure S8, Supporting Information) and analyzed using an R1‐(R2‐C1) equivalent circuit. The ionic resistances of pristine CG, CG@C and CG@CP were determined to be 8.11, 11.83, and 8.99 Ω, respectively. The values of resistances and conductivities are listed in Table S2 of the Supporting Information. It is known that interlayers usually restrict the transport of ions.^[^ ^13^ ^]^ A decreased ionic conductivity was observed for CG@C separator. By contrast, after CP coating, the ionic conductivity remained almost the same as with the blank separator, which implies that the Mo_6_S_8_ coating layer will not impede the ion transport in the cells. In addition, CP itself is an electrochemical active component based on the redox reaction Mo_6_S_8_↔Mg_x_Mo_6_S_8_ and may have relatively low impact on capacity‐reduction at cell level.

A model S/C composite was used as cathode material and was prepared by a melt‐diffusion method at 160 °C.^[^ ^14^ ^]^ Typical X‐ray diffraction (XRD) patterns in Figure S9a of the Supporting Information show the broadening of the S diffraction peaks with much reduced intensity for the S/C composite compared to the pure elemental sulfur, indicating amorphous sulfur was deposited inside the pores of carbons. Thermogravimetric analysis (TGA) and differential scanning calorimetry measurement of the S/C composite in argon flow revealed a weight loss of ≈60% at 700 °C, which corresponds to the evaporation of sulfur in the composite (Figure S9b, Supporting Information). It is noteworthy to mention that the sulfur loading of the cathode material is higher than those of the reported Mg–S batteries.^[^ ^8^, ^12^, ^15^ ^]^ Besides, SEM/EDS analysis of the morphology and element distribution of the cathode material manifests that sulfur was uniformly dispersed within the carbon host material (Figure S9c–e, Supporting Information).

**Figure 3:**
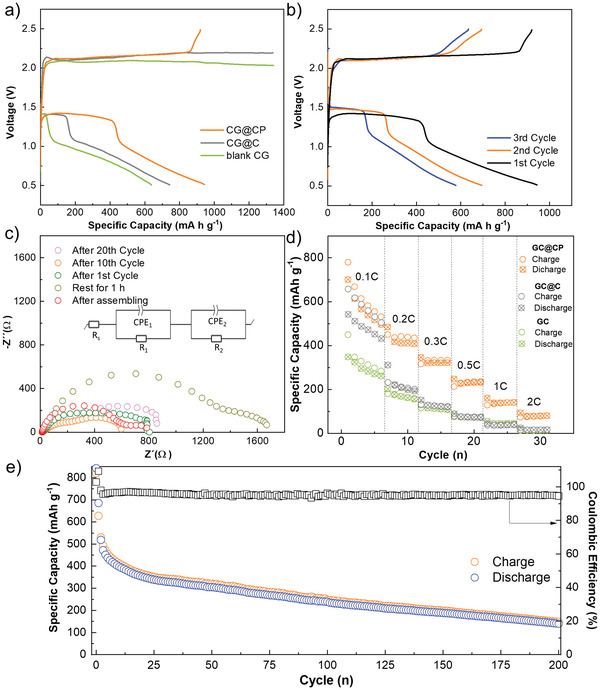
Galvanostatic discharge/charge voltage profiles of Mg–S cells with a) CG@CP, CG@C, and pristine CG in the first cycle and b) CG@CP in first 3 cycles at a current density of 0.1 C. c) Nyquist plots of the Mg–S cell with CG@CP after different cycles. d) Rate performance of Mg–S cells with different separators at a current density at 0.1 C, 0.2 C, 0.3 C, 0.5 C, 1 C, and 2 C. e) Long cycling performance of Mg–S cells with CG@CP at a current density of 0.2 C.

Galvanostatic discharge/charge profiles of Mg–S cells with CG, CG@C or CG@CP are displayed in **Figure** **3**a,b. A long and flat voltage profile at 2.1–2.2 V during charge in the first cycle was observed when using CG and CG@C, indicating the severe polysulfide shuttle behavior.^[^ ^10^, ^16^ ^]^ By contrast, no obvious overcharging was detected in the cell with CG@CP, and the system delivered a high discharge capacity of 942.9 mA h g^−1^ in the 1st cycle. More detailed electrochemical performance of cells with pristine CG and CG@C are presented in Figures S10 and S11 of the Supporting Information. With pristine CG, the cell achieved discharge capacities of 638 and 457 mA h g^−1^ in the 1st and the 10th cycle, respectively, and failed during the 12th cycle. It is worth to point out that the active material loss caused by the dissolution of polysulfide has a detrimental influence on the cycle life of Mg–S cells.^[^ ^17^ ^]^ By contrast, with CG@C, the initial discharge capacity of the cell increased to 740 mA h g^−1^ and showed a capacity recovery during the first 3 cycles, probably due to the coated carbon layer which may adsorb certain amount of polysulfides. However, the cells broke down also fast. EIS measurements were carried out to determine the resistances of layers formed inside cells with different separators. In the Nyquist plots, the depressed semicircles include two parts: a nonblocking interface contact resistance (*R*_int_) in the high frequency region, as represented by R_1_//CPE_1_ in the fitting circuit and a high charge transfer resistance (*R*_ct_) in the low frequency region, corresponding to R_2_//CPE_2_. Detailed fitting parameters are shown in Table S3 of the Supporting Information. After the 1st cycle, the *R*_int_ of cell with CG@CP decreased from 231.9 to 130.1 Ω, after 20 cycles to 3.5 Ω. While with the pristine CG, *R*_int_ after rest was relatively high at 887.0 Ω and stayed at 446.3 Ω after the 1st cycle. The *R*_int_ value in the cell with CG@C stands between the cells with CG and CG@CP. Meanwhile, *R*_ct_ of the cell with CG increased incrementally with cycling and reached 1896.0 Ω after 20 cycles. The continuous increasing *R*_ct_ is mainly due to the reduced electrochemical kinetics upon prolonged cycling. With CG@CP and CG@C, the values of *R*_ct_ were inevitably growing with cycle number, but a much lower resistance was obtained with CG@CP (1030.0 Ω). These results support the hypothesis that CP can bind the polysulfides to maintain a stable interface of electrode and synergistically accelerate the redox kinetics. The significantly enhanced kinetic was further validated by the rate performance of the Mg–S cells in Figure 3d. The specific capacity of the cell with CG@CP was continuously decreasing at 0.1 C and became relatively stable from 0.2 C with a capacity ≈417 mA h g^−1^. In comparison, the discharge capacities of cells with CG@C and pristine CG at 0.2 C were 215 mA h g^−1^ and 171 mA h g^−1^, respectively, indicating relatively sluggish kinetics of sulfur conversion and less efficient sulfur utilization. Long cycling tests were performed with CG@CP at 0.2 C (Figure 3e). Notably, the cell was able to maintain stable cycling for 200 cycles with a Coulombic efficiency of 96% (capacity retention ≈ 150 mA h g^−1^). These results manifest that the coating layer is beneficial for capturing the polysulfide species and simultaneously promoting its conversion, thus minimizing the loss of the active material, avoiding the passivation of anode and accelerating battery kinetics.

The EDS point analysis for the CG@CP before and after cycling is demonstrated in Figure S12 of the Supporting Information. Before cycling, the atomic ratio of S/Mo was 1.27 (close to 1.33, the stoichiometric ratio of Mo_6_S_8_) while it increased to 1.59 after cycling, which indicates the adsorption of MgS_n_ or S_n_^−^ on the coated separator. At the anode side, the results of elemental analysis of Mg foil from the cells with different separators after 5 cycles are summarized in Table S4 of the Supporting Information. On the Mg foil collected from the cell with CG, sulfur already appeared after 5 cycles. With the CP‐coated separator, there was no sulfur signal detected on the anode side, which confirms the effective polysufide suppression of the Mo_6_S_8_ layer. The XRD patterns and EDS maps of CG@CP before and after cycling indicate that both the composition and morphology of the Mo_6_S_8_ coating layer were maintained (Figures S13 and S14, Supporting Information).

From the discharge/charge voltage profiles of Mg–S cells with CP‐coated separator, no capacity contribution from Mo_6_S_8_ could be obviously observed. To further explore the capacity contribution of Mo_6_S_8_, coin cells were assembled with CP@CG and Mg anode. The Mo_6_S_8_ layer contacts the cathode cap and works as cathode. Galvanostatic charge/discharge experiments were performed in a voltage range of 0.5–2.5 V versus Mg/Mg^2+^ and at a current of 167.5 µA (corresponding to 0.1 C with 1 mg sulfur as active material) as shown in Figure S15 of the Supporting Information. It demonstrated that Mo_6_S_8_ layer can contribute an increasing specific capacity with cycling and stabilize at about 17 mA h g^−1^.

Apart from using the Mo_6_S_8_ modified separator, a hybrid cathode (Mo_6_S_8_ mixed with S/C) was also tried. As reported in Li–S cell,^[^ ^18^ ^]^ by combining intercalation‐type Mo_6_S_8_ with conversion‐type sulfur, both high gravimetric and volumetric energy densities are able to be delivered simultaneously. The XRD pattern of the hybrid cathode material and the electrochemical performance are shown in Figure S16 of the Supporting Information. Unfortunately, with the hybrid cathode, the cells still showed an evident overcharging behavior in the first cycle and a rapid break down. In this case, the Mo_6_S_8_ in the cathode composite did not show obvious effect of inhibiting the polysulfide shuttle, which may be ascribed to the limited contact between Mo_6_S_8_ and sulfur species inside the cathode. As reported, the polysulfide shuttle consumes mainly the sulfur close to the separator.^[^ ^9^, ^19^ ^]^ By contrast, the dissolved magnesium polysulfide can easily access the Mo_6_S_8_ particles on separator, suggesting that the concept of modified separator is more efficient approach for polysulfide suppression and migration.

**Figure 4:**
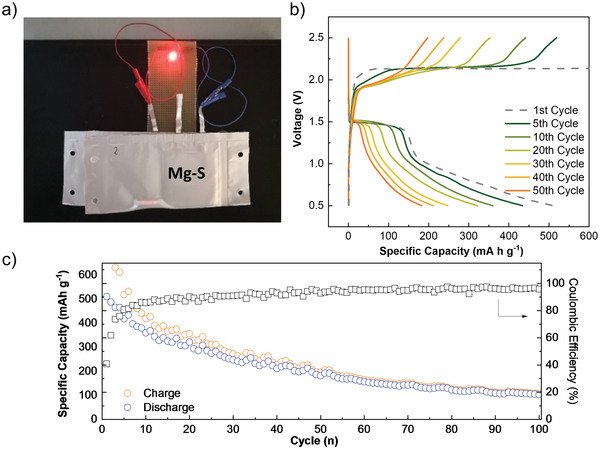
a) Illustration of LED lighted by Mg–S two pouch cells with CG@CP. b) Galvanostatic voltage profiles of Mg–S pouch cell with the 1st, 5th, 10th, 20th, 30th, 40th, and 50th cycles. c) Cycling performance of Mg–S pouch cell at a current density of 0.05 C.

To further validate the integration of the functional separator in a practical cell configuration, lab‐scale pouch cells were successfully assembled in a format of 58 × 58 mm^2^ as shown in **Figure** **4**a. The mass loading of sulfur on the cathode was 0.5 mg cm^−2^. Figure 4b displays the galvanostatic discharge/charge profiles of the pouch cell. The specific capacity value was calculated based on the mass of sulfur in the composite. An initial discharge capacity of 512 mA h g^−1^ was delivered in the first cycle. The followed (first) charge process presented a long charge plateau (Figure S17, Supporting Information) with a low initial Coulombic efficiency. From the second cycle, the overcharge plateau was significantly shortened with a continuously increased Coulombic efficiency, and the Coulombic efficiency maintained over 90% after 20 cycles. Moreover, the pouch cell remained a reversible capacity of 182 mA h g^−1^ after 50 cycles and 103 mA h g^−1^ with a Coulombic efficiency of 95% for the 100th cycle (Figure 4c). To the best of our knowledge, there are few reports about the performances of Mg–S cells or Mg ion cells in pouch cell configuration.^[^ ^9^, ^20^ ^]^ With functional separator the Mg–S pouch cell showed an extend lifespan up to 100 cycles, which offers valuable insight into the feasibility of the composites for full‐scale battery application. Besides, the unit cost required for the pouch cell is also inexpensive, considering the low cost of both electrodes^[^ ^21^ ^]^ and less amount of Mo_6_S_8_ on the functional separator (0.2–0.3 mg cm^−2^). The results further confirm that the functional separator can be used on a large‐scale application for developing metal–sulfur systems.

### 2.4 Catalytic Effect of Mo_6_S_8_ in the Mg–S Cell

**Figure 5:**
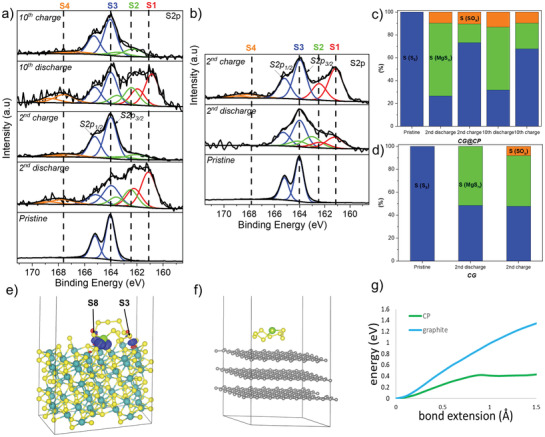
a,b) S 2p XPS spectra of the S/C cathodes facing CP‐coated Celgard (CG@CP) and Celgard (CG) separator. c,d) Relative atomic percentage of S‐containing compounds on the surfaces of S/C cathode in function to separators (CG@CP vs CG) after cycling. Charge transfer between MgS_8_ and e) Chevrel phase or f) graphite upon adsorption. Blue areas indicate places with increased electron density, while red areas indicate places with decreased electron density. g) Energy change during the scan of the bond S2–S3 of MgS_8_ adsorbed at CP and graphite.

To gain more insight into the mechanism of CP, XPS measurements were conducted on the S/C cathodes after the 2nd cycle (charge/discharge) and the 10th cycle (charge/discharge) as shown in **Figure** **5**. S 2p spectra reveal four chemical environments of sulfur on the S/C cathodes after cycling. The S4 doublet (S 2p_3/2_: 166–168 eV) is the signal of sulfides oxidation species (SO_x_), which have often been observed on the cathode surfaces after cycling and is irrelevant to the analysis.^[^ ^22^ ^]^ The signal of the elemental sulfur is represented by S3 doublet (S 2p_3/2_: 164 eV). Two additional doublets S2 and S1 whose S 2p_3/2_ peaks located ≈162.5 eV (green) and 161 eV (red) are attributed to MgS and terminal sulfur atoms from MgS_n_ polysulfide (MgS_n_–T). According to Figure 5a,b, the S 2p spectra of both cathodes after the 2nd discharge reveal the dominating presence of polysulfide (MgS_n_–T) and MgS signals. After the 10th cycle, the cathode using CG@CP separator shows the same properties as that after two cycles, revealing a high reversibility. By contrast, with CG separator, after the 2nd charge, high percentages of the sulfur signal are attributed to MgS_n_, indicating a poor reversibility of the oxidation reaction from MgS*_n_* to sulfur. The results were also confirmed with ex situ Raman measurements (Figure S18, Supporting Information). After the 2nd charge cycle, the cathode from the cell with modified separator shows the Raman bands with high intensity at 150, 219, and 470 cm^−1^ corresponding to sulfur and possibly S_8_ rings as reported before.^[^ ^23^ ^]^ While with the cathode from another cell with CG separator, there is no obvious S_8_ Raman band. Besides, the cell with CG separator often failed at around the 10th cycle with variable sulfur signals. These results confirm that CG@CP allows a promoted reversibility of the sulfur redox chemistry and better capacity retention upon cycling.^[^ ^24^ ^]^

The interaction of the magnesium polysulfides with CP, was further studied with DFT calculations. Taking MgS_8_ as an example, a noteworthy transfer of negative charge toward magnesium polysulfide was detected (Figure 5e). The additional charge is mainly located around magnesium cation and two sulfur atoms of S3 and S8. On the other hand, no alteration in the electron density was observed during adsorption of MgS_8_ on the carbon surface, indicating a weak interaction (Figure 5f). To clarify the effect of the presented electron transfer on the polysulfide conversion reaction, the decomposition process of the S_8_^2−^ anion into S_6_^−^ and S_2_^−^ was simulated by the breaking of the bond between the 2nd (S2) and 3rd (S3) sulfur atom, as we expect this S–S to be the weakest from the analysis of observed electron transfer. During the stretching of the S2–S3 bond, there was a large difference between two studied surfaces (Figure 5g). When passing from graphite to the CP, the energy needed to break the S–S bond was reduced by more than 1 eV, which shows that Mo_6_S_8_ can work as a strong mediator and enhance the kinetics of the conversion from the high‐order polysulfide toward the low‐order polysulfide. This reduction in the barrier during the S2–S3 bond breaking in the CP is due to an additional increase of the charge transfer to S2 and S3 atoms, which is not observed when the substrate is graphite (Figure S19, Supporting Information). Similar analysis for another three reactions involving polysulfides with an even number of S atoms were also conducted (Figure S20, Supporting Information). The energies needed to extend the S–S bond by 1.5 Å are summarized in Table S5 of the Supporting Information, which can clearly indicate the improved kinetics by replacement of carbon to CP material. In addition, an analysis of the chemical dissociation energy for MgS and MgS_8_ was also conducted to study the supporting role of Chevrel phase. The energies needed to separate the cation and anions at CP, 0.17 and 0.32 eV, respectively, are much lower than for the analogues processes at the carbon surface, 1.06 and 0.55 eV, respectively (Figure S21, Supporting Information). Therefore, the reduced energy barrier on Mo_6_S_8_ facilitates the conversion reaction, indicating the catalytic effects on the reaction kinetics of Mg–S systems.

## 3 Conclusion

In summary, a modification of separator has been presented with Mo_6_S_8_ functional layer via a facile slurry‐casting process, which can mitigate the polysulfide shuttle, enhance the conversion of polysulfide and significantly improve the performance of the Mg–S cells in terms of reversible discharge capacity and cycle‐life (≈200 cycles). XPS analysis and DFT calculations revealed the dual effect of Mo_6_S_8_ on promoting polysulfide conversion and inhibiting polysulfide shuttle in Mg–S batteries. Additionally, the prototype Mg–S pouch cells further confirm the feasibility of the functional separator for full cell application. The study highlights the beneficial effects of an electrochemically active interlayer on cell performance and may offer a new pathway for the development of practical rechargeable Mg–S batteries.

## 4 Experimental Section

### Preparation of S/C Cathode and Mg Anode

S/C composite was prepared by a commonly used melt‐diffusion method.^[^ ^14^ ^]^ Typically, 0.4 g of Ketjen Black (KB) and 0.6 g of sulfur powder mixture were ball‐milled under Ar for 2 h, and heated to 160 °C for 20 h under Ar atmosphere. The cathode electrode was prepared by casting 80 wt% active material (S/C), 10 wt% SuperP (Sigma‐Aldrich), and 10 wt% poly(vinyl difluoride) (PVDF) with *N*‐methylpyrrolidinone (Sigma‐Aldrich) onto a one side carbon‐coated Al foil. The loading mass of the active material was ≈0.6–0.8 mg cm^−2^.

When preparing the hybrid cathode, 10 wt% of Mo_6_S_8_ was added by casting. Mg foil (0.1 mm, Gelon Energy Corp) was tailored into proper disks with a diameter of 14 mm. Before being used as Mg anode, these Mg disks were polished carefully in a glovebox.

### Preparation of Coated Separator

Typically, 60 wt% Mo_6_S_8_ powder (NEI Corporation, USA), 30 wt% SuperP, and 10 wt% PVDF were mixed as slurry. Subsequently, the homogeneous slurry was cast onto one side of Celgard separator (Celgard 2340, PP/PE/PP, 38 µm thick) as shown in Figure 4a. The dried CG@CP was punched into disks with a diameter 16 mm. The mass loading of the Mo_6_S_8_ was ≈0.2–0.3 mg cm^−2^ with a thickness of 20 µm. The carbon‐coated separator was prepared in the same way with 90 wt% SuperP and 10 wt% PVDF.

### Electrolyte Synthesis

Commercially available anhydrous diethyl ether and dimethoxy ethane (DME, Sigma) were stored over 3 Å molecular sieves in glovebox for at least 24 h prior to use. Hexafluoroisopropanol ((CF_3_)_2_CHOH, 99%, Alfa Aesar) was dried over 3 and 4 Å mixed molecular sieves. NaBH_4_ (98%, Sigma‐Aldrich) and anhydrous MgCl_2_ (99%, Sigma‐Aldrich) were used as received to synthesis Mg(BH_4_)_2_. The magnesium tetrakis(hexafluoroisopropyloxy)borate (Mg[B(hfip)_4_]_2_) electrolyte was synthesized in a reaction between Mg(BH_4_)_2_ and hexafluoroisopropanol in DME as reported.^[^ ^11c^ ^]^ 0.3 м electrolyte solution was prepared by dissolving proper amount of magnesium salt in DME. And the concentration is based on the molecular weight of Mg[B(hfip)_4_]_2_^.^3DME.

### Preparation of Magnesium Polysulfide (MgS*_n_*) Solution

2.053 g (64.0 mmol) of sulfur powder and 0.194 g (8.0 mmol) of Mg powder was ball‐milled at 200 rpm for 10 h using silicon nitride vial and balls under Ar atmosphere. Transfer the powder material to a glass vial in glove box and 30 mL of tetraglyme was added. The suspension was then stirred at 60 °C for 3 days. Finally, the suspension was filtrated and the reddish MgS_n_ solution was used for adsorption experiments.

### Pouch Cell Assembly

To assemble pouch cells, the S/C electrode sheets with dimensions 50 × 50 mm^2^ were used as cathodes. Mass loading of sulfur was 0.5 mg cm^−2^, on one side of the electrode. On the anode side, Mg foil was punched into proper sheets with the dimension of 54 × 54 mm^2^. Between the electrodes, a modified Celgard separator and an additional piece of borosilicate glass fiber (Whatman, GF/C) were added in the size of 58 × 58 mm^2^. The pouch cells were assembled in a dry room at a dew point of −50 °C and the same electrolyte was used as for the coin cells. The cells were discharged to 0.5 V at 0.05 C after resting for 10 h.

### Characterizations

The XRD measurements were conducted on Bruker‐AXS D8 diffractometer using a Cu K*α* X‐ray source in the range of 10° to 80° with a step size of 0.02°. Thermal analysis of the samples was carried out with TGA coupled with differential scanning calorimetry in a Setaram thermal analyzer of a SENSYS evo instrument. The measurement was conducted from room temperature to 700 °C under synthetic argon flow with a heating rate of 5 °C min^−1^. SEM images were obtained using a ZEISS LEO 1530 at 10 kV electron beam with EDS. The SEM samples were prepared on carbon tape followed by gold sputtering. The XPS spectra were acquired using a Thermo Scientific K‐alpha spectrometer. The samples were analyzed using a microfocused, monochromated Al K*α* X‐ray source (1486.6 eV, 400 µm spot size). XPS spectra were recorded with a concentric hemispherical analyzer at a pass energy of 50 eV and fit with one or more Voigt profiles (binding energy uncertainty: ±0.2 eV) and Scofield sensitivity factors were applied for quantification^[^ ^25^ ^]^ using the Advantage software package. All S/C cathode spectra were referenced to the S 2p peak (S–S from S_8_) at 164.0 eV binding energy controlled by means of the photoelectron peaks of metallic Cu, Ag, and Au, respectively. The sulfur (S 2p) spectra were done at the beginning and after each resolution analysis, to check absence any sample degradation under irradiation. Raman spectra were collected at room temperature in the spectral range of 100–800 cm^−1^ using a laser with a wavelength of 532 nm and laser power of 2.5 mW as the excitation source.

### Electrochemical Measurements

The CR2032 coin cells and three‐electrode cells (PAT‐Cell, EL‐CELL) were assembled in an argon‐filled glove box (H_2_O, O_2_ ˂0.1 ppm). The cells were discharged to 0.5 V after resting for 1 h. Except for the modified or blank Celgard separator, an additional piece of borosilicate glass fiber separator was also added at the same time. EIS was carried out on an electrochemical workstation (VMP3 Biologic) from 1 MHz to 10 mHz with a DC voltage amplitude of 10 mV. For Mg–S cells, galvanostatic charge/discharge experiments were performed in a voltage range of 0.5–2.5 V versus Mg/Mg^2+^ and at a current density of 0.1 C or 0.2 C (1 C = 1675 mA g^−1^) with an Arbin battery cycling unit. Cyclic voltammetry measurements were carried out with a scan rate of 0.1 mV s^−1^ in a voltage range of 0.5–2.8 V versus Mg/Mg^2+^. All electrochemical investigations were done under 25 °C.

### Theoretical Calculations

All DFT electronic structure calculations were performed using Vienna ab initio Simulation Package. All calculations employed the Perdew–Burke–Ernzerhof exchange‐correlation functional and projector augmented wave potentials for all elements. An energy cut‐off of 520 eV was imposed for the plane‐wave basis. All surfaces were generated based on optimized bulk geometries, and consisted of 2 or 3 layers, respectively for Chevrel phase and graphene materials. The optimization of surfaces was performed fixing the positions of the atoms in the bottom layer, to account for the contact of the surface with bulk. To simulate contact of upper layers with electrolyte, 30 Å of vacuum was introduced and implicit solvation model was employed with dielectric constant set to be equal to 7.4, corresponding to ethereal solvents.^[^ ^26^ ^]^ On such prepared surfaces, molecules of MgS, MgS_2_, MgS_4_, MgS_6_, MgS_8_, and S_8_ were placed in at least five random locations each, and optimized in order to find the global minimum. Only the lowest energy geometries found were used for the study. The interaction energy was calculated as a difference between the energy of the surface with adsorbed specie and the sum of the energies of the pristine surface and the isolated adsorbate specie. To assess the stability of the S–S bond in MgS_8_ adsorbed at graphite and Mo_6_S_8_, the bond scan was performed by extending the length of the S2–S3 bond by 0.1 Å at each step and optimizing the rest of the structure. To speed up the scan calculations, the size of the surfaces was reduced by removal of the bottom layer. All calculations were performed using a Gamma‐centered 3 × 2 × 1 k‐mesh grid, and the convergence criteria were set to 0.02 eV Å^−1^. The charge assignment to the atoms was performed using Bader Charge Analysis. For adsorbed MgS and MgS_8_ species, the dissociation energy was calculated as a difference between the structure of adsorbed ion‐pair and adsorbed separated ions with a distance of around half of unit cell (6 Å).

## Conflict of Interest

The authors declare no conflict of interest.

## Supporting information

Supporting Information

## Data Availability

The data that support the findings of this study are available from the corresponding author upon reasonable request.
