# Supplementary material for: Dual Role of Mo6S8 in Polysulfide Conversion and Shuttle for Mg–S Batteries
Source: Adv Sci (Weinh). 2022 Jan 9;9(7):2104605. doi: 10.1002/advs.202104605 (PMC8895118; doi:10.1002/advs.202104605)
Supplement: Supplementary file 1 — Supporting Information [file ADVS-9-2104605-s001.pdf]

WILEY-VCH

ADVANCED SCIENCE
Open Access

Supporting Information

for *Adv. Sci.,* DOI: 10.1002/advs.202104605

Dual role of Mo_6S_8 in polysulfide conversion and shuttle for Mg–S batteries

Liping Wang, Piotr Jankowski, Christian Njel, Werner Bauer, Zhenyou Li, Zhen Meng, Bosubabu Dasari, Tejs Vegge, Juan Maria García Lastra, Zhirong Zhao-Karger* and Maximilian Fichtner**

Supporting Information

Dual role of Mo_6S_8 in polysulfide conversion and shuttle for Mg–S batteries

Liping Wang, Piotr Jankowski, Christian Njel, Werner Bauer, Zhenyou Li, Zhen Meng, Bosubabu Dasari, Tejs Vegge, Juan Maria García Lastra, Zhirong Zhao-Karger* and Maximilian Fichtner**

The details of XPS:

Concerning Mo 3d spectra they have to be fitted with $3d_{5/2}$-$3d_{3/2}$ doublet separated by 3.2 eV with 3/2 intensity ratio due to spin-orbit coupling. In addition, the sulfur (S 2s) environments already observed and described in S 2p spectra, Figure 2d reveals four kinds of molybdenum environments. The Mo1 asymmetric doublet (228.1-231.3eV) can be attributed to Mo-Mo bond.[1] The Mo2 (229.1-232.3eV) and Mo3 (229.7-232.9eV) doublets can be attributed to the molybdenum oxidation states Mo^{2+} and Mo^{3+}, respectively. These peaks are characteristic to oxidation states of molybdenum in molybdenum sulfides structures.[2] The fourth doublet (Mo4) located at 232.5-235.8eV can be assigned to Mo^{6+} oxidation state characteristic to Molybdenum in oxygen environments such as MoO_3.[3] This peak disappears after immersion that suggests that is due to the sample surface oxidation from cross-contamination. The Mo1, Mo2 and Mo3 peaks are the signature of the Mo_6S_8 structure characterized in this study. Their presence after immersion in MgS_n solution confirms the chemical stability of CG@CP in the presence of polysulfides.

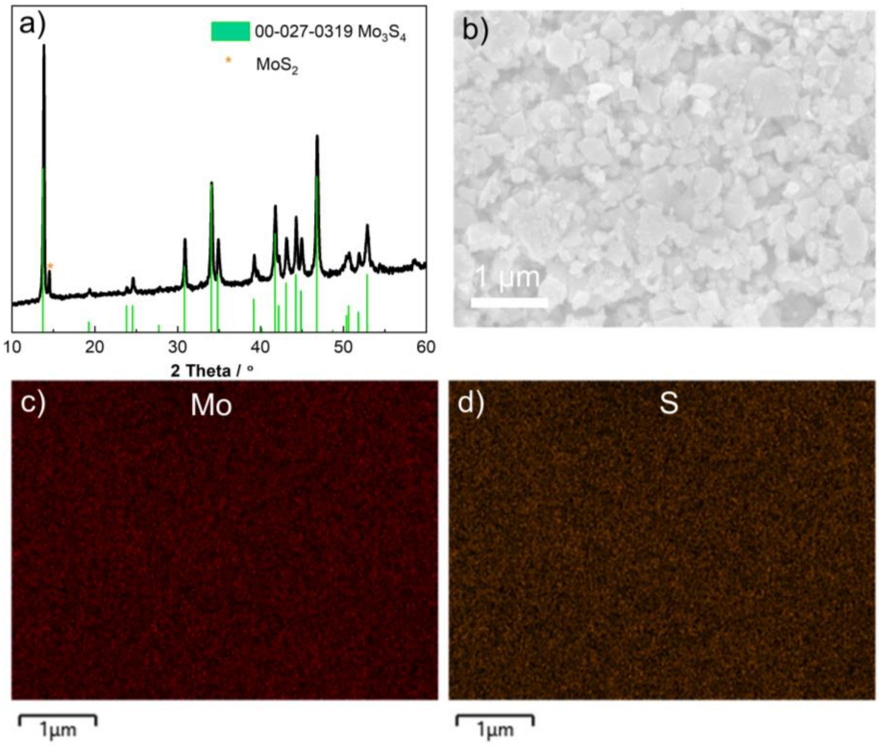

Figure S1. (a) Typical XRD pattern of Mo_6S_8 material. (b-d) SEM images and EDS maps of Mo_6S_8 material.

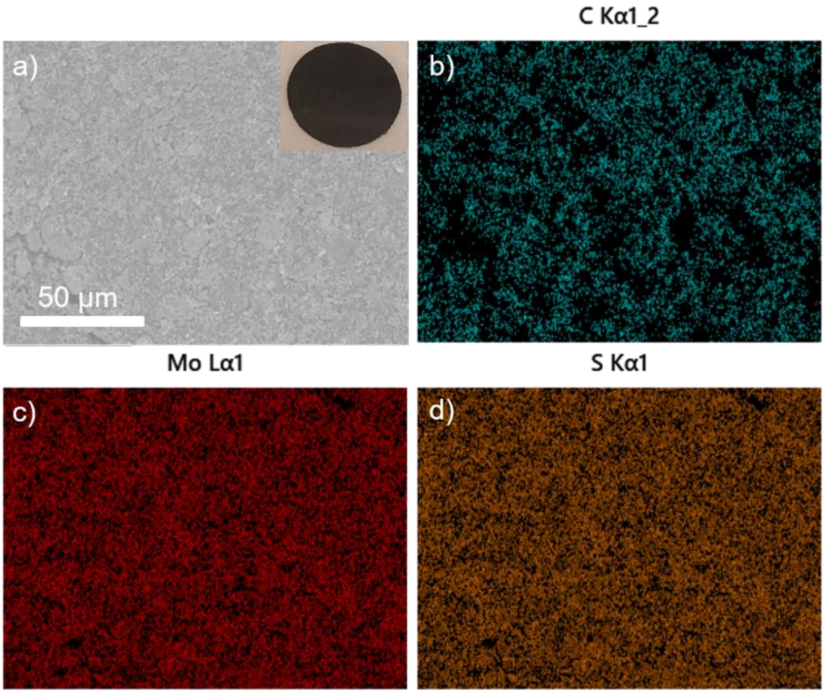

Figure S2. SEM image and EDS maps of CG@CP ((a) Inset: the photograph of CG@CP).

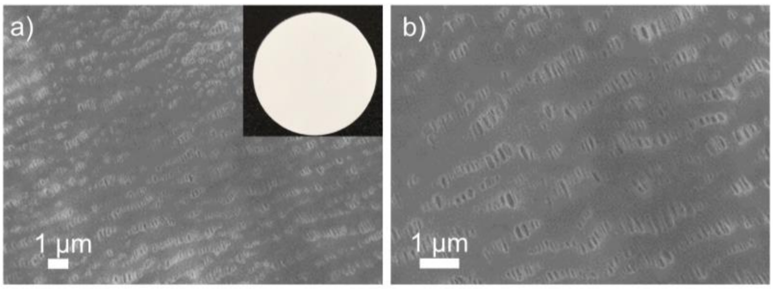

Figure S3. SEM images of pristine CG ((a) Inset: the photograph of CG).

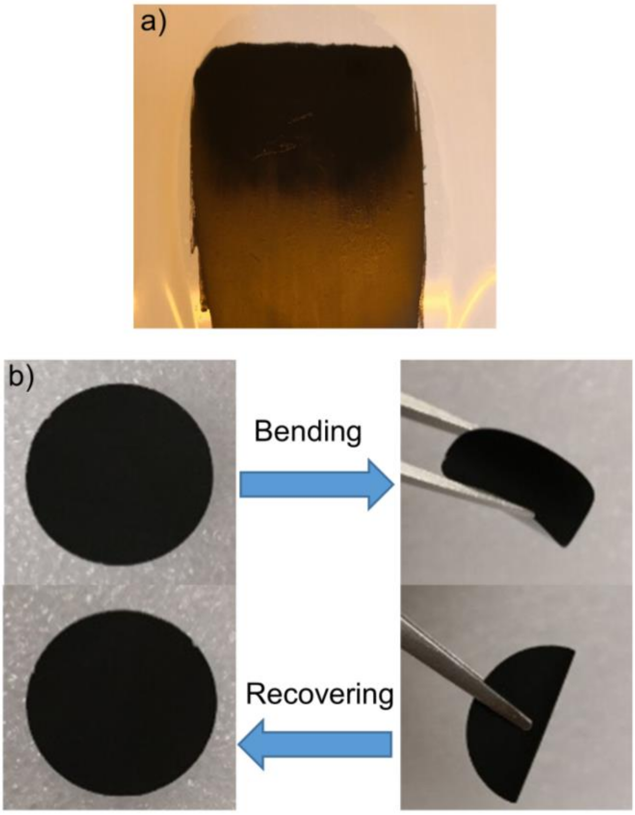

Figure S4. Photographs of (a) slurry coated separator and (b) CG@CP separator at bending state and after recovery.

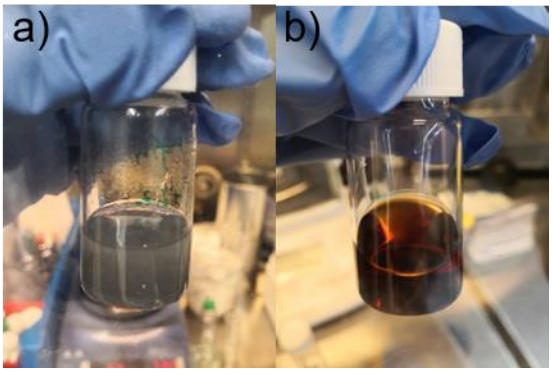

Figure S5. (a) Photograph of the mixture of Mg and sulfur powder in tetraglyme solution before stirring. (b) MgS_n in tetraglyme solution.

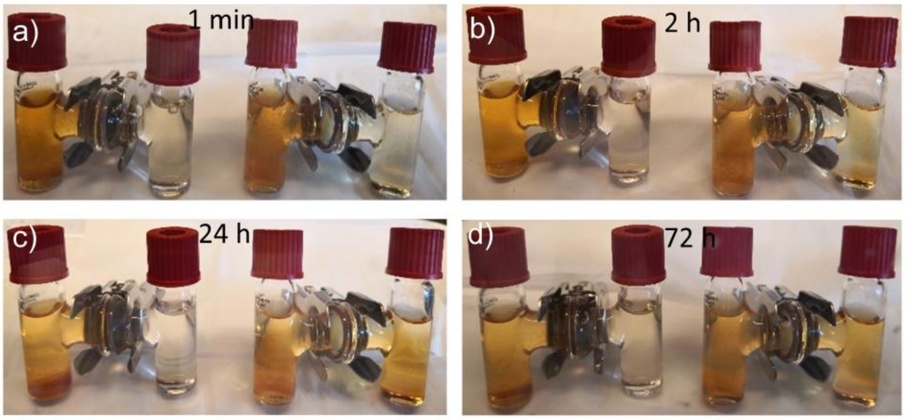

Figure S6. Optical images of the diffusion of polysulfides: visualized H-type glass devices with a pristine glass fiber separator (right) and a GPE filled separator (left). The tetraglyme solvent with MgS_n was injected in the left chamber and the solvent without polysulfides was injected in the right side.

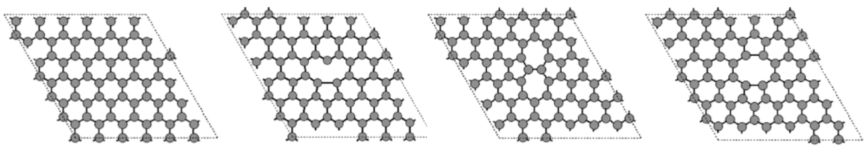

**S_8**	2.12 eV	2.09 eV	2.12 eV	2.06 eV
**MgS_8**	1.00 eV	0.93 eV	1.08 eV	0.91 eV
**MgS_6**	0.59 eV	0.54 eV	0.61 eV	0.57 eV
**MgS_4**	0.37 eV	0.42 eV	0.47 eV	0.50 eV
**MgS_2**	0.42 eV	0.60 eV	0.50 eV	0.72 eV
**MgS**	0.31 eV	0.48 eV	0.28 eV	0.37 eV

Figure S7. Adsorption energies of S_8 and MgS_n at different surfaces of graphite, considering ideal layer and containing three different defects.

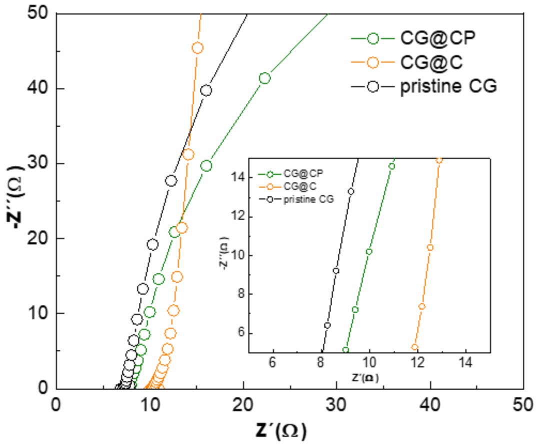

Figure S8. EIS results of SS//CG or CG@C or CG@CP//SS cell at 25°C.

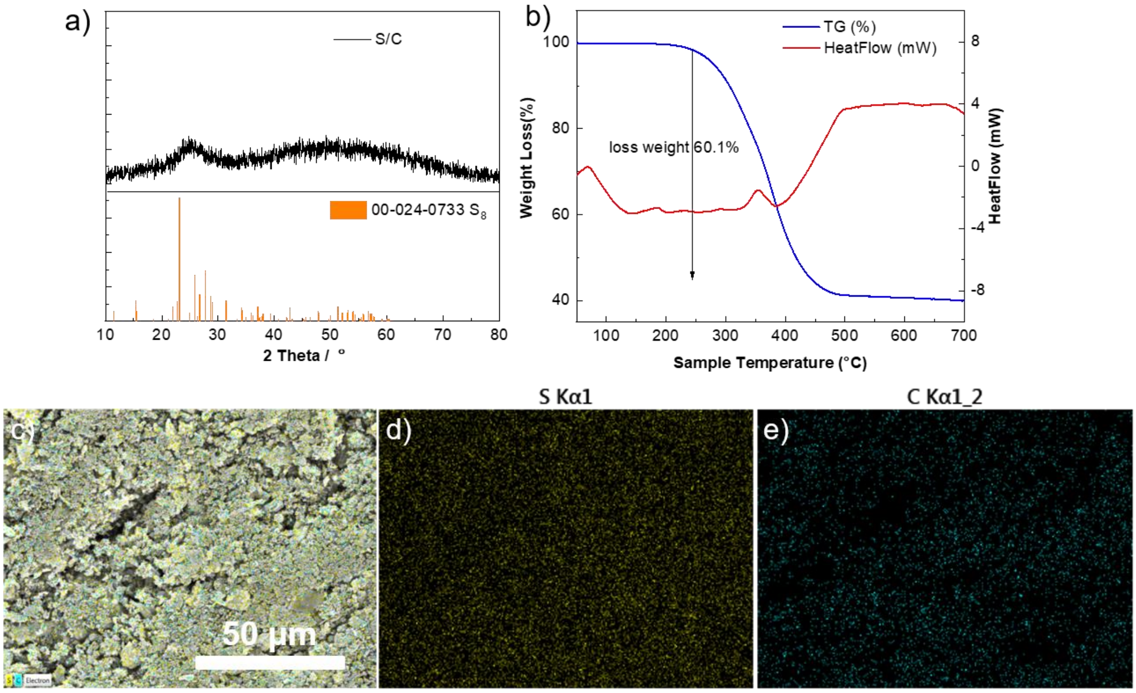

Figure S9. (a) Typical XRD patterns and (b) TGA and DSC analysis of S/C cathode material. (c–f) SEM images and EDS maps of S/C cathode plates.

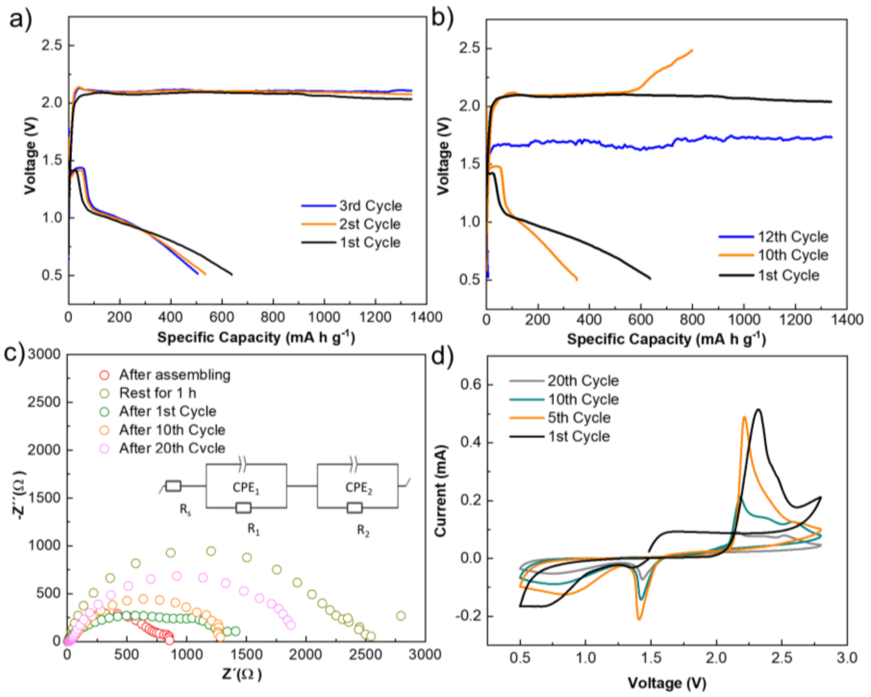

Figure S10. Galvanostatic discharge/charge voltage profiles of Mg–S cell with CG (a) in the first three cycles and (b) in the 1st, 10th and 12th cycles at a current density of 0.1C. (c) Nyquist plots of the Mg–S cell with CG after different cycles. (d) Cyclic voltammetric profiles at the scan rate of 0.1 mV s^{-1} with the 1st, 5th, 10th and 20th cycles.

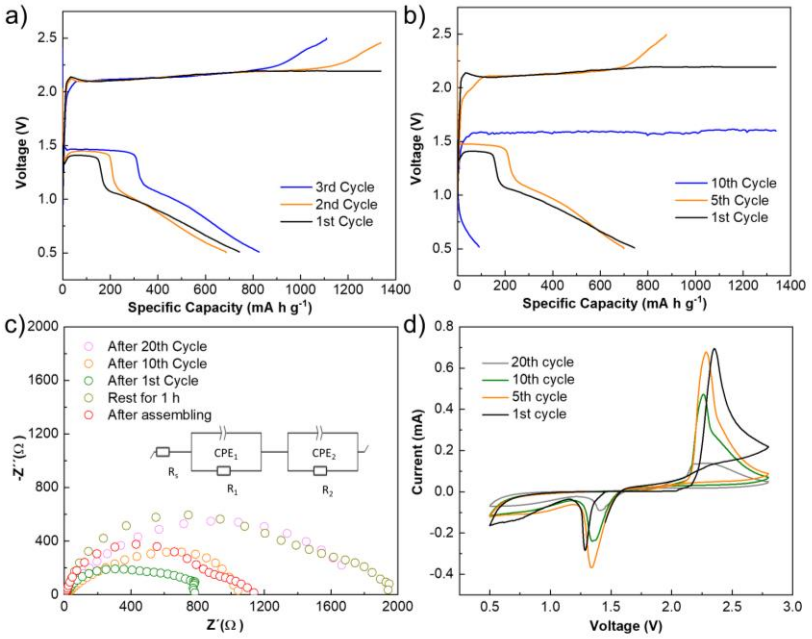

Figure S11. Galvanostatic discharge/charge voltage profiles of Mg–S cell with CG@C (a) in the first three cycles and (b) in the 1st, 5th and 10th cycles at a current density of 0.1C. (c) Nyquist plots after different cycles. (d) Cyclic voltammetric profiles at the scan rate of 0.1 mV s^{-1} with the 1st, 5th, 10th and 20th cycles.

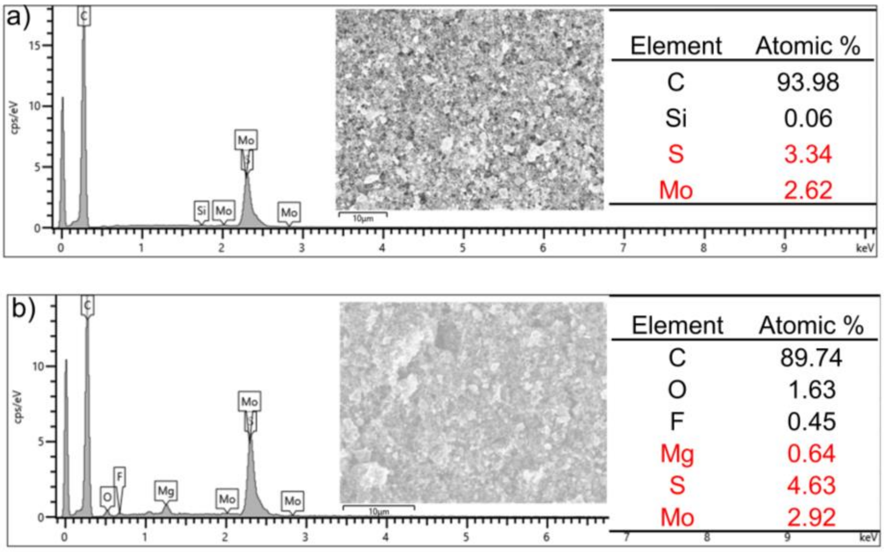

Figure S12. EDS point analysis for CG@CP (a) before and (b) after 20 cycles. (Inset: Corresponding SEM image and atomic concentration from EDX point analysis)

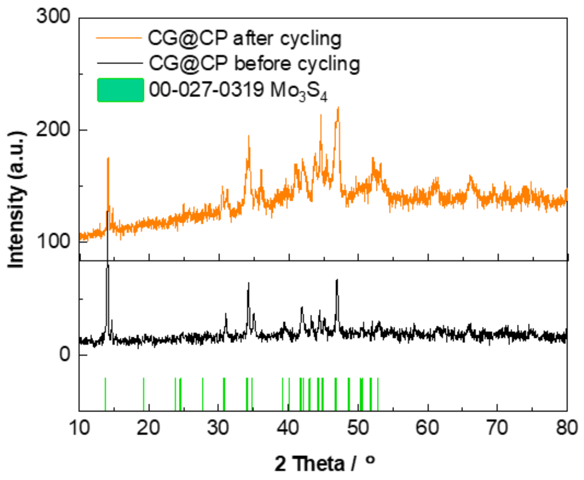

Figure S13. Typical XRD pattern of CG@CP before and after cycling.

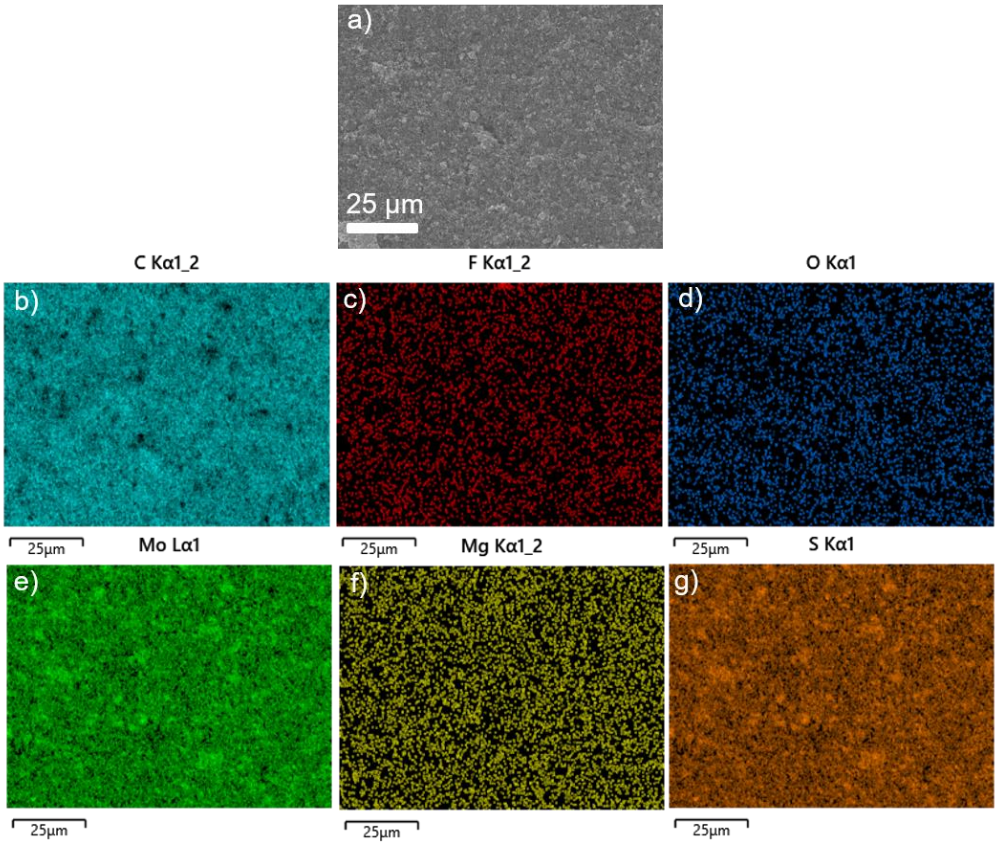

Figure S14. SEM image and EDS maps of CG@CP after 20 cycles.

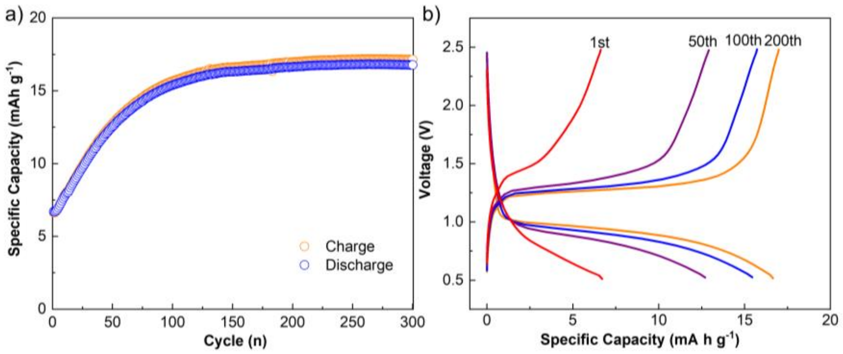

Figure S15. Galvanostatic discharge/charge voltage profiles of cell with CG@CP (a) in the 1st, 50th, 100th and 200th cycles at a current of 167.5 µA. (b) Long cycling performance of cells with CG@CP at a current of 167.5 µA.

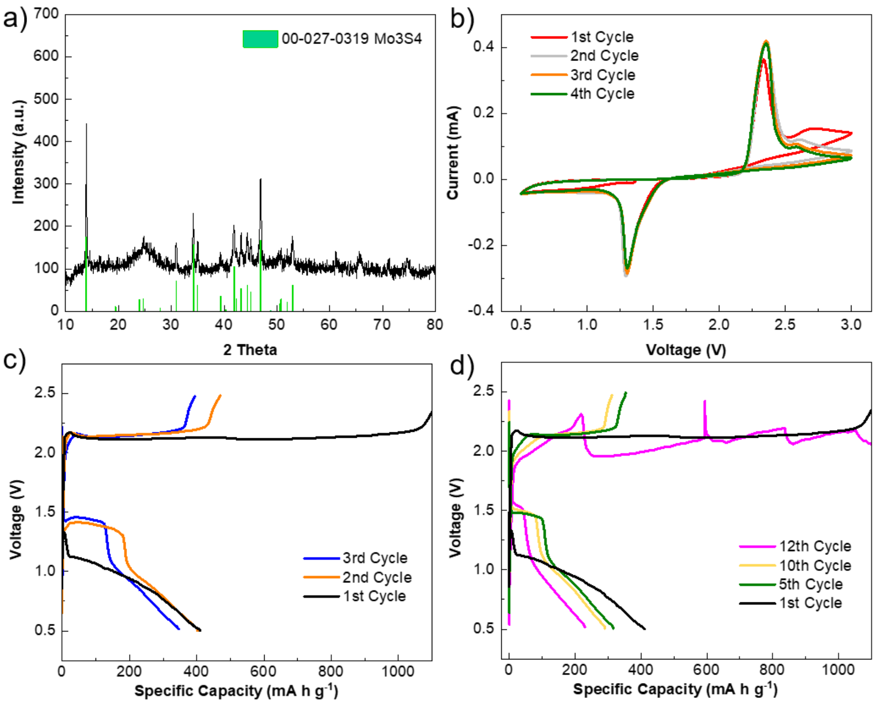

Figure S16. (a) Typical XRD pattern of hybrid cathode material. (b) CV profiles of Mg–S cells with hybrid cathode at the scan rate of 0.1 mV s^{-1} for the first four cycles. Galvanostatic discharge/charge voltage profiles of Mg–S cells (c) in the first three cycles and (d) in the 1st, 5th, 10th and 12th cycles.

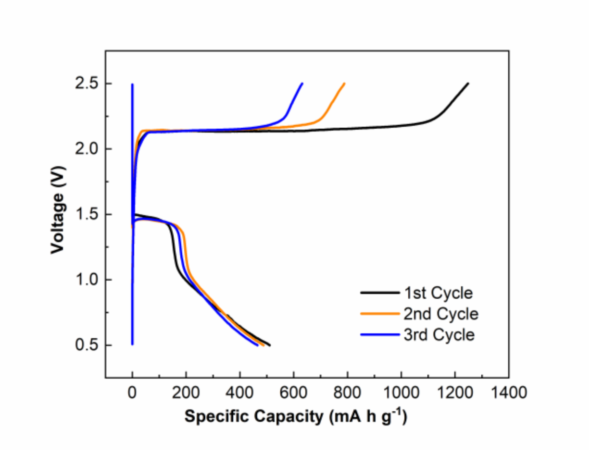

Figure S17. Galvanostatic discharge/charge voltage profiles of Mg–S pouch cell in the first three cycles.

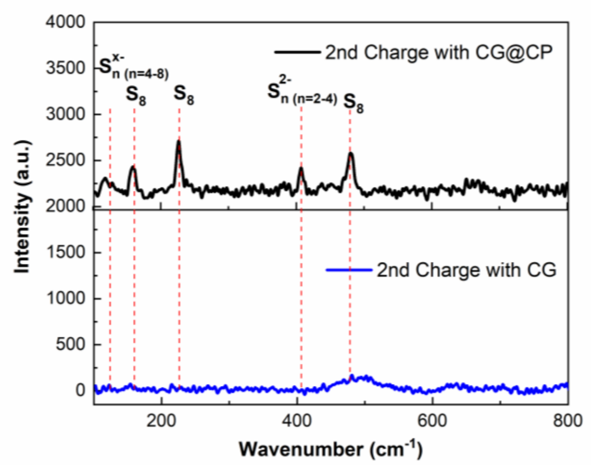

Figure S18. *Ex-situ* Raman spectra of the S/C cathode in Mg/S cells with different separators after the 2nd charge cycle.

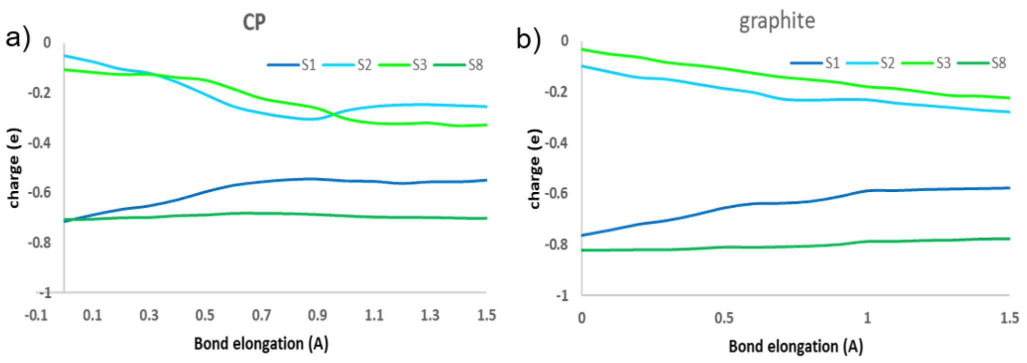

Figure S19. Evolution of charges at selected sulfur atoms of MgS_8 molecule adsorbed at (a) Chevrel phase or (b) graphite during S2-S3 bond elongation.

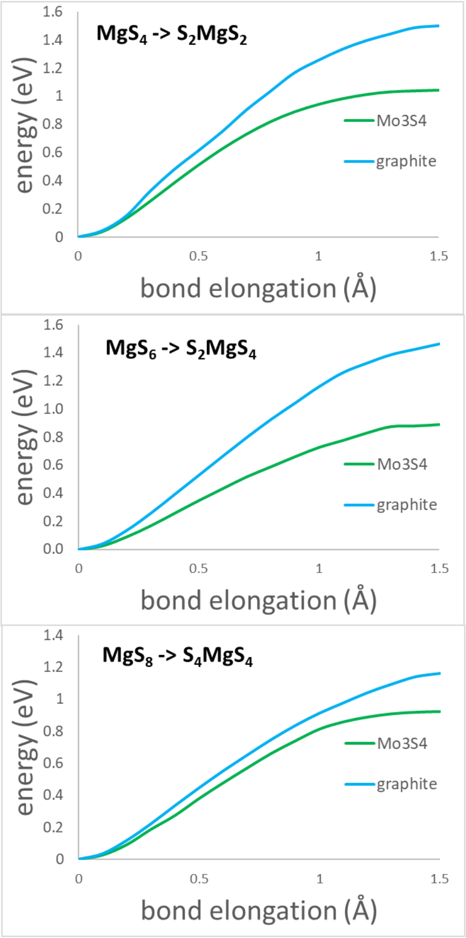

Figure S20. Energy change during the scan of different bonds of MgS_n adsorbed at CP and graphite: bond S2-S3 of MgS_4 (top), bond S2-S3 of MgS_6 (middle), and bond S4-S5 of MgS_8 (bottom).

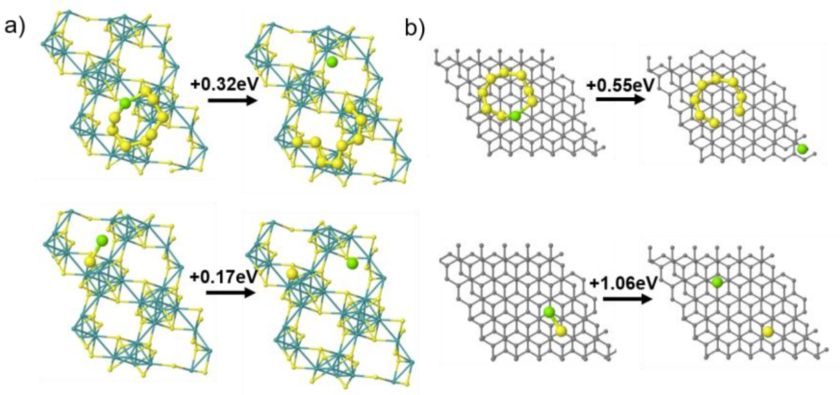

Figure S21. Analysis of MgS_8 and MgS dissociation process at the surfaces of (a) Chevrel phase and (b) graphite. Energies indicate the thermodynamic difference between the shown structures.

Table S1. Interaction energy between surface of Chevrel phase material at different stages of magnesation and sulfur/magnesium polysulfides

Eint/eV	Mo_6S_8	$MgMo_6S_8$	$Mg_2Mo_6S_8$
S_8	2.83	2.69	2.56
MgS_8	2.93	2.40	2.20
MgS_6	2.69	2.42	2.25
MgS_4	2.43	2.17	2.13
MgS_2	2.88	2.31	2.21
MgS	3.10	2.43	2.19

Table S2. Summary of the ionic conductivities of CG, CG@C and CG@CP

Materials	R_i / Ω	σ_i / S cm^{-1}
CG	8.11	$3.53 \cdot 10^{-4}$
CG@C	11.83	$2.42 \cdot 10^{-4}$
CG@CP	8.99	$3.19 \cdot 10^{-4}$

Ionic conductivities were calculated according to equation 1:

$$\sigma = l / (R_i \cdot A) \quad (1)$$

Where l is the thickness of the separator (0.0038 cm), A is the cross-sectional area (1.327 cm^2) and R is the resistance.

Table S3. Summary of fitting parameters for various EIS plots of batteries with different separators

	R	Rest for 0 h	Rest for 3 h	After 1 cycle	After 10 cycles	After 20 cycles
	R_s/Ω	3.0	2.6	3.7	3.8	4.4
CG	R_{int}/Ω	369.3	887.0	446.3	80.8	33.1
	R_{ct}/Ω	530.5	1992.0	846.1	1229.0	1896.0
	R_s/Ω	7.8	6.8	6.7	6.4	8.3
CG@C	R_{int}/Ω	197.5	421.3	221.2	445.4	7.1
	R_{ct}/Ω	883.2	1496.0	563.0	683.9	1802.0
	R_s/Ω	12.3	15.3	16.4	17.0	17.5
CG@CP	R_{int}/Ω	162.6	231.9	130.1	171.7	3.5
	R_{ct}/Ω	585.9	1432.0	698.2	423.2	1030.0

Table S4. Weight percentage concentration from EDS analyses of Mg anodes in cells with different separators

Element (Wt%)	CG	CG@C	CG@CP
C	12.10	20.65	14.19
O	24.53	19.10	14.00
F	11.63	19.84	9.44
Mg	49.08	40.41	62.37
S	2.66	0.00	0.00

Table S5. Energy needed to break selected S-S bond by extension of the bond by 1.5 Å on the surface of Chevrel Phase and graphite.

E/eV	Mo_6S_8	graphite
$MgS_8 \rightarrow S_2MgS_6$	0.43	1.34
$MgS_8 \rightarrow S_4MgS_4$	0.92	1.16
$MgS_6 \rightarrow S_2MgS_4$	0.89	1.46
$MgS_4 \rightarrow S_2MgS_2$	1.04	1.50

References

[1] T. Kaewmaraya, M. Ramzan, J. M. Osorio-Guillén, R. Ahuja, *Solid State Ionics* **2014**, 261, 17.
[2] J. Richard, A. Benayad, J. F. Colin, S. Martinet, *J. Phys. Chem. C* **2017**, 121, 17096.
[3] J. C. Dupin, D. Gonbeau, I. Martin-Litas, P. Vinatier, A. Levasseur, *Appl. Surf. Sci.* **2001**, 173, 140.
